# Health-Related Quality of Life in Pulmonary Hypertension and Its Clinical Correlates: A Cross-Sectional Study

**DOI:** 10.1155/2018/3924517

**Published:** 2018-03-19

**Authors:** Abílio Reis, Mário Santos, Margarida Vicente, Inês Furtado, Célia Cruz, Alzira Melo, Luísa Carvalho, Fabienne Gonçalves, Pedro Sa-Couto, Luís Almeida

**Affiliations:** ^1^Pulmonary Vascular Disease Unit, Medicine Department, Centro Hospitalar do Porto, Hospital de Santo António, Porto, Portugal; ^2^Cardiology Service, Medicine Department, Centro Hospitalar do Porto, Hospital de Santo António, Porto, Portugal; ^3^Department of Physiology and Cardiothoracic Surgery, Faculty of Medicine of Porto, Porto, Portugal; ^4^Department of Health Sciences, University of Aveiro, Aveiro, Portugal; ^5^Internal Medicine Service, Medicine Department, Centro Hospitalar do Porto, Hospital de Santo António, Porto, Portugal; ^6^Center for Research and Development in Mathematics and Applications, Department of Mathematics, University of Aveiro, Aveiro, Portugal; ^7^MedInUP, Department of Pharmacology and Therapeutics, Faculty of Medicine, University of Porto, Porto, Portugal

## Abstract

**Background:**

Health-related quality of life (HRQoL) impairment is common in pulmonary hypertension (PH), but its clinical predictors are not well established. This study aims to characterize the HRQoL of patients with pulmonary arterial hypertension (PAH) and other precapillary forms of PH (pcPH) and to explore its clinical correlates.

**Materials and Methods:**

A cross-sectional, observational study of patients with documented PAH and other forms of pcPH. Patients completed two patient-reported outcome measures (PROM): Cambridge Pulmonary Hypertension Outcome Review (CAMPHOR) and Nottingham Health Profile (NHP). Clinical characteristics were retrieved from electronic medical records.

**Results:**

Mean CAMPHOR and NHP scores for the study population were indicative of a moderate HRQoL impairment. Patients in World Health Organisation Functional Classes (WHO FC) III/IV showed significantly worse HRQoL. The main clinical correlates of HRQoL were WHO FC, 6-minute walking distance (6MWD), and Borg dyspnoea index. Overall quality of life (QoL), assessed through CAMPHOR's QoL domain, showed patterns comparable to HRQoL measured by both instruments.

**Conclusions:**

HRQoL, measured by two different PROMs, is impaired in Portuguese patients with PAH and other forms of pcPH, particularly in patients with increased disease severity. WHO FC, 6MWD, and Borg dyspnoea index are highly correlated with HRQoL and QoL.

## 1. Introduction

Pulmonary hypertension (PH) encompasses a vast group of chronic and progressive disorders characterized by an increase in pulmonary artery pressure, which can be associated with a variety of aetiologies [1]. If left untreated, PH can cause right ventricular failure and, ultimately, death [2–5]. In clinical practice, several severity and prognosis indicators are usually assessed during patient diagnostic workup and management, including the World Health Organisation functional class (WHO FC), 6-minute walking distance (6MWD), Borg Dyspnoea Index, and several laboratory biomarkers, which include invasive hemodynamic evaluation [6–9]. However, these indicators do not provide a direct estimation of the overall health status and quality of life (QoL). Therefore, in recent years, there has been an increasing interest in measuring both health-related quality of life (HRQoL) and QoL using general or specific-disease patient-reported outcome measures (PROMs) [10].

Defining the concepts of health status, HRQoL, and QoL is yet a matter of controversy among experts, which is well expressed in the medical literature [11]. This leads to the lack of a standardised terminology, which can introduce substantial interpretation issues. So, every scientific investigation in this field should start by clarifying the criteria used to define such concepts and, at the same time, by characterizing the properties and capacities of the instruments used to evaluate the populations under study. For the purpose of this study, we define health status as the narrower of the three concepts, including all aspects of physical, mental, and social functioning that characterize an individual at a given time. HRQoL, on the other hand, evaluates the effects of the physical, mental, and social aspects—and particularly the effects of illness and treatment—on the individual's sense of well-being. QoL is the broader of the three concepts covering all aspects of life, including non-health-related aspects such as economic status and social participation, to characterize an individual's overall sense of well-being.

In populations with PH, both HRQoL and QoL have been assessed through various instruments over the years. Initially, general scales such as the Short-Form 36 (SF-36) or the Nottingham Health Profile (NHP) were the most widely used to assess HRQoL; these instruments did not, however, allow complete QoL evaluation. Recently, a PH-specific instrument, the Cambridge Pulmonary Hypertension Outcome Review (CAMPHOR) [12], was developed and validated for use in various regions, both in clinical practice and in clinical research settings [13]. CAMPHOR was the first PH-specific instrument to be developed and it provided substantial advantages over previously used instruments. It includes targeted evaluation of PH-specific symptomatology and activities that allows a better characterization of HRQoL in the PH population and includes an additional domain specifically aimed at assessing overall QoL. CAMPHOR was carefully developed and validated for use in PH populations with its content derived directly from patients and, thus, it was widely used over the past decade and is currently validated for use in several countries [12, 14–17]. More recently, other PROMs, like emPHasis-10 [18] and PAH-SYMPACT® [19], have also been developed to allow adequate collection of patient-reported information on health status, but without the capacity to actually evaluate QoL. Therefore, CAMPHOR continues to play and important role in PH, since it is the only PROM that integrates measures of QoL and provides an actual measure of patient value.

Given the nature of the physiological changes that characterize PH, this condition is expected to impact patient functionality, HRQoL, and QoL. Actually, several studies have identified HRQoL impairment in varied PH populations [20]. Although the relationships between patient's clinical characteristics and HRQoL impairment were not fully elucidated, some clinical factors seem to be highly associated with these outcomes, including WHO FC, 6MWD, symptoms (dyspnoea, fatigue, chest pain), drugs and administration route, and mental health (anxiety and depression) [2, 20–24]. Still, given the infrequent nature of precapillary forms of PH and the difficulties in studying these populations, there is still a need for further data on this field.

On the other hand, although we have a significant number of studies with various PROMs in these populations [20], there is yet a lack of scientific evidence about the value of the retrieved information and about the most efficient and easy to administer ones. This study aims to characterize the HRQoL of patients with pulmonary arterial hypertension (PAH) and other precapillary forms of PH (pcPH) assessed by a general instrument (NHP) and a disease-specific instrument (CAMPHOR), to evaluate HRQoL impairment and to explore the correlations between clinical characteristics and HRQoL measured through these PROMs and, ultimately, to give insights into the value of such questionnaires in the global evaluation of patients with these highly debilitating conditions.

## 2. Materials and Methods

### 2.1. Study Design

This is a cross-sectional, observational study of consecutive patients with documented PAH and other forms of pcPH (confirmed by right heart catheterisation) followed at a specialised PH unit at a tertiary care centre in Northern Portugal (Pulmonary Vascular Disease Unit, Medicine Department, Centro Hospitalar do Porto, Porto, Portugal). During the process of CAMPHOR validation for the Portuguese PH population, patients were asked to complete two questionnaires aimed at assessing their HRQoL (CAMPHOR and NHP) and to complete a basic questionnaire on their demographic and clinical characteristics. Disease-specific clinical measures, including haemodynamic ones, were retrieved from the hospital electronic medical records (EMR).

The study received favourable opinion from the Ethics Committee of Centro Hospitalar do Porto (Porto, Portugal). The study protocol and data collection instruments were submitted and approved by the Portuguese National Data Protection Commission. All patients provided their written informed consent prior to inclusion in the study.

### 2.2. Patient Population

Patients were eligible to participate in the study if they were ≥18 years old and were able and willing to give their informed consent. Patients were excluded if they were unable to complete the study questionnaires due to illiteracy or cognitive impairment or if their medical records revealed a medical condition or circumstance that could compromise their ability to comply with the study protocol.

### 2.3. Data Collection and Instruments

HRQoL data were collected by self-administering questionnaires during a scheduled routine clinical visit. Participants were asked to complete 3 questionnaires: (1) CHAMPOR, (2) NHP, and (3) a general demographic/clinical questionnaire, which evaluated age, gender, race, income, education level, and self-reported length of PH diagnosis. Clinical and laboratory data were retrieved from the database of the dedicated EMR software of the Unit (PAHTool®, Inovultus Ltd., Santa Maria da Feira, Portugal).

### 2.4. Cambridge Pulmonary Hypertension Outcome Review (CAMPHOR)

CAMPHOR was the first questionnaire specifically validated for PH patients, designed to assess symptoms, functioning, and QoL in clinical practice and in clinical trials [12].

CAMPHOR consists of (1) a 25-item overall symptoms scale, scored 0–25, with higher scores indicating the presence of more PH symptoms; (2) a 15-item functioning scale, scored 0–30, with lower scores indicating good functioning; and (3) a 25-item quality of life scale, scored 0–25, with higher scores indicating poor QoL [12]. The QoL subscale was developed using the needs-based model [25, 26]; that is, it is based on the premise that QoL is derived from the ability of the individual to satisfy his/her needs [12].

The symptom and quality of life scales have dichotomous response options (“true”/“not true” or “yes”/“no”) while the functioning scale has three-point response options (“able to do on own without difficulty”/“able to do on own with difficulty”/“unable to do on own”).

### 2.5. Nottingham Health Profile (NHP)

NHP is a questionnaire that allows the assessment of the general health status of a given population, which can be used to assess HRQoL. NHP comprises 38 items, which fall into six sections: energy level (3 items), pain (8 items), emotional reactions (9 items), sleep disturbance (5 items), social isolation (5 items), and physical mobility (8 items) [27]. Individual items are scored 1 for a “yes” response and 0 for a “no” response. The total score for each section represents the summation of item scores expressed as a percentage. For each section, scores range from 0 to 100, with higher scores representing greater perceived distress (i.e., impaired health status) [27].

### 2.6. Statistical Analysis

Descriptive data are presented as mean ± SD or frequency (%). Bivariate analysis correlating demographic and clinical variables with CAMPHOR/NHP dimensions scores was conducted using Spearman's Rank correlation coefficient (between quantitative variables) and using point-biserial correlation (between quantitative variables and binary nominal variables). Multiple linear regression analysis was conducted only for the significant correlations to identify possible demographic/clinical predictors for both CAMPHOR and NHP scales. This technique was chosen due to the quantitative nature of the dependent variables. Significant variables were selected using a stepwise approach (stepping method criteria: entry, 0.05; removal, 0.10) and no estimation problems were found. A dummy variable technique was used to incorporate qualitative independent variables in the regression models. The assumption of residual's normality for the multiple regressions was verified visually by inspection of the PP plot.

Statistical analyses were conducted with SPSS Statistics for Windows, version 23.0 (IBM Corp, Armonk, NY, USA), and results were considered significant if *P* < 0.05.

## 3. Results

### 3.1. Study Population Characteristics

A total of 49 patients accepted to participate in the study and completed the study questionnaires (*N* = 49).  Table 1 summarises the demographic and clinical characteristics of the study population. Mean ± SD age was 50.4 ± 13.7 years and most patients were female (75.5%). Mean disease duration was 57.1 ± 58.8 months. One or more comorbidities were present in 57.1% of patients. The most common PH aetiologies were chronic thromboembolic pulmonary hypertension (CTEPH) (24.5%), congenital heart disease (22.4%), idiopathic/heritable (22.4%), connective tissue disease (14.3%), and others (16.3%). Others included PAH-associated portal hypertension and HIV (3 and 1 patients) and 4 patients with group 5 PH. To allow meaningful statistical analysis, PH aetiologies are, from this point on, categorised as PAH and others (including Group 1 PH and Group 5 PH) and CTEPH (Group 4 PH).

Most patients had PH disease markers compatible with low (28.6%) or intermediate (53.0%) estimated risk of 1-year mortality, according to the 2015 ESC/ERS guidelines risk assessment scale [1]. Most patients were in WHO FC I and II (69.4%). Mean 6MWD was 428 ± 105.8 meters in the overall population, but it was significantly reduced in patients in WHO FC III/IV (320.3 ± 99.4 meters) compared to groups I/II (469.1 ± 75.4 meters; *P* < 0.001).

An arterial oxygen desaturation (94.0 ± 3.4 to 80.5 ± 15.1%) during the 6-MWT was found as well as a moderate elevation of N-terminal pro-brain natriuretic peptide (NT-proBNP) levels (668.6 ± 908.4 *μ*g/mL). Most of patients (87.7%) were under PH-specific therapy, 46.9% in monotherapy and 53.0% in combination therapy. Specific therapy was predominantly administered through oral route (73.5%). A substantial number of patients were under oxygen therapy (40.1.%).

### 3.2. Health-Related Quality of Life Assessed through CAMPHOR and NHP

**Figure 1:**
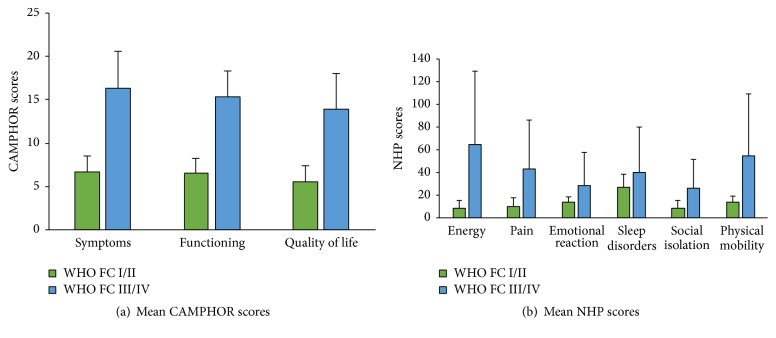
Mean CAMPHOR and NHP scores according to WHO Functional class. CAMPHOR: Cambridge Pulmonary Hypertension Outcome Review; NHP: Nottingham Health Profile; WHO FC: World Health Organisation functional class. Error bars represent 95% confidence intervals. Please note that the upper limit for NHP score is 100; confidence intervals are shown here for illustration purposes.

Overall, the study population showed mean CAMPHOR scores that were indicative of a moderate HRQoL impairment: symptoms (9.6 ± 7.7); functioning (9.3 ± 6.3); quality of life (8.1 ± 7.0). Mean NHP scores were also indicative of a moderate health-related quality of life impairment: energy level (27.0 ± 37.2), pain (20.3 ± 31.1), emotional reactions (18.2 ± 18.6), sleep disturbance (30.2 ± 34.6), social isolation (13.9 ± 24.2), and physical mobility (26.3 ± 26.2). Importantly, mean scores for both CHAMPOR and NHP were significantly worse in patients in WHO FC III/IV, compared to WHO FC I/II (Figure 1). In patients with WHO FC III/IV, all CAMPHOR domains showed significantly higher scores, whereas for NHP the domains showing the worse results were energy level, pain, and physical mobility. In terms of overall QoL, as measured through the QoL domain of CAMPHOR, patients showed comparable patterns, with scores indicative of a moderate impairment of QoL. CAMPHOR and NHP scores according to gender, PH aetiology, oxygen therapy, and PH-specific therapy are explored in Supplementary Materials (available here).

### 3.3. Clinical Correlates of Health-Related Quality of Life

Table 2 explores the relationship between patient demographic and clinical characteristics and CAMPHOR scores. In bivariate analysis, WHO FC, 6MWD, and Borg dyspnoea index were highly correlated with all CAMPHOR domains (correlation >0.5 or <−0.5, with *P* < 0.001); scatterplots for high correlations are shown in Supplementary Materials. Other factors such as age, oxygen use, baseline and maximum heart rate, and oxygen saturation were also significantly correlated with CAMPHOR scores, but with correlations of lower magnitude. In multivariate analysis only 6MWD and Borg dyspnoea index were consistently associated with CAMPHOR scores. Interestingly, for the functioning dimension of CAMPHOR, in multivariate analysis, WHO FC and baseline heart rate were statistically significant factors, whereas Borg dyspnoea index was not a significant factor for this dimension only.

Table 3 explores the relationship between patient demographic and clinical characteristics and NHP scores. The relationships between NHP scores and patient characteristics were highly variable between the different dimensions. In bivariate analysis, WHO FC, 6MWD, and Borg dyspnoea index were highly correlated with the energy level, pain, and physical mobility domains of NHP. However, for the emotional reactions and social isolation domains, Borg dyspnoea index and baseline heart rate were the more relevant factors in terms of bivariate correlation. Also, the sleep disorders dimension only showed significant correlation with gender, Borg dyspnoea index, baseline heart rate, and use of combination therapy, but with correlations of lesser magnitude. In multivariate analysis, WHO FC and 6WMD remained significantly associated with NHP scores in the energy level and physical mobility domains. For the pain domain the significant factors were PH aetiology and maximum heart rate, for the emotional reactions domain the significant factors were Borg dyspnoea index and baseline heart rate, for sleep disorders the significant factor was only gender, and for social isolation the significant factor was only the Borg dyspnoea index. Nonetheless, linear regression models showed lower predictive value (*R*^2^ < 0.3) for the pain, sleep disorders, and social isolation domains (Table 3). Interestingly, clinically relevant factors such as age, PH aetiology, disease duration, comorbidities, conservative therapy (including oxygen therapy), or PH-specific therapy did not show particularly strong correlations with CAMPHOR or NHP scores, especially in multivariate analyses.

## 4. Discussion

This study characterized the HRQoL of a cohort of Portuguese PH patients with mostly low to intermediate risk of estimated 1-year mortality (low disease severity), using two parallel questionnaires to provide a more comprehensive assessment of overall patient status. HRQoL was moderately impaired for the majority of this PH population. Increased disease severity, assessed by WHO FC, was significantly associated with greater impairment. Patients in WHO FC classes III and IV showed significantly higher scores for all the dimensions of the general and disease-specific instruments, indicative of important HRQoL impairment.

The PH population included in this study had low disease severity (approximately 70% of patients in WHO FC I/II), despite having a mean disease duration over 50 months. This low disease severity can be explained by the fact that most patients were under combination PH-specific therapy at of the time of the study. When compared with other studies that used NHP and CAMPHOR to assess HRQoL in PH, our population had substantially lower disease severity. While most studies included approximately 70% of patients in WHO FC III/IV [28–32], we included 70% of patients on the other end of the spectrum (WHO FC I/II). Such a low profile of disease severity was only previously reported by one study assessing HRQoL through CAMPHOR in a population of patients receiving PH-specific treatment [33]. This patient profile of low disease severity and mortality risk is an important distinctive factor for this study, especially in the current context in which continued treatment innovations will lead to better treatment outcomes in the future. In a context of improved vital and clinical outcomes, improving QoL will be a major treatment goal, thus, studies that evaluate QoL in low disease severity populations can play an important role in establishing better strategies to assess patient outcomes.

CAMPHOR scores in this study were numerically lower than those reported in previous studies [28–31, 33], but in some cases the differences are very small, even below 1 point in the CAMPHOR scores. Lower scores are attributable to low disease severity in this study population. Furthermore, the significantly higher scores showed by patients in WHO FC III/IV were comparable to previous findings [31], which further validates this relationship between CAMPHOR scores and disease severity.

In this study mean NHP scores were particularly high for the dimensions of energy level, sleep disturbance, and physical mobility (ranging from 25–30%). Scores were also elevated for pain and emotional reactions (approximately 20%). Together these findings indicate an overall impairment of HRQoL. A previous study that used the NHP in a PH population with more severe disease (75% in WHO FC III/IV) reported substantially higher scores for all NHP dimensions [32]; NHP scores in our subpopulation of patient in WHO FC III/IV were comparable to the findings of that study, which also supports a relationship between disease severity and NHP scores.

In terms of clinical correlates of HRQoL, the 6MWD and Borg dyspnoea index were the two factors with stronger association with CAMPHOR scores, including scores for the QoL domain. WHO FC was also shown to be a highly relevant factor in bivariate analysis, but since it is intrinsically related to exercise capacity and dyspnoea, in multivariate analysis it only reached statistical significance in the functioning domain (where Borg dyspnoea index was not a significant factor). These findings largely agree with previous evidence that found these three measures to be the most relevant predictors of HRQoL in PH [2, 23, 24]. NHP scores showed a more variable relationship with clinical correlates, which was somewhat expected since NHP evaluated various aspects of patients' life in different dimensions. WHO FC and 6MWD remained the more important correlates for the physical functioning dimensions (energy level and physical mobility), but for the remaining dimensions linear regression models had substantially lower predictive value, which could indicate that variables relevant for these dimensions were not considered in this study. We hypothesise that some measures of mental health status could have been important for inclusion in these models, since other studies have identified conditions such as depression and anxiety to be correlated with HRQoL in PH [21, 22].

This study has limitations inherent to the relatively small sample size and the less severe disease stage of the studied patients. Nonetheless, the study is based on high-quality clinical data, systematically and prospectively collected using a purpose-designed EMR (PAHTool®) at a PH specialised unit, which further ensures data reliability. Additionally, there are also limitations associated with the instruments used to assess HRQoL. Although we used both a general and a disease-specific instrument to provide a more comprehensive picture of overall patient status, other instruments (especially general instruments) could also have been selected. In the future, HRQoL should be evaluated in large Portuguese PH populations (eventually in multicentre studies to attain larger and more diverse samples), employing other highly used general instruments (such as the SF-36 or EQ50) as well as the recently developed disease-specific instruments (emPHasis-10 and PAH-SYMPACT). Characterizing HRQoL with these new instruments in larger and more heterogeneous populations (in terms of severity) will provide an important basis for clinical practice assessments and for comparisons with results from clinical trials. Still, the value of CAMPHOR as the only disease-specific instrument capable of evaluating overall QoL should not be understated. While other recently developed instruments might prove valuable to the clinician, they are only designed to assess HRQoL and, therefore, do not demonstrate actually patient value; this can only be established with an instrument tailored to evaluate QoL, such as the CAMPHOR. This study highlights the importance of CAMPHOR as disease-specific instrument of choice when evaluating HRQoL and QoL in PH patients. It also highlights some aspects of patients' lives that are not fully captured by CAMPHOR (such as mental status) and which should be considered during HRQoL and QoL assessments.

In conclusion, HRQoL is impaired in Portuguese patients with PAH and other forms of pcPH, particularly in patients with increased disease severity. General (NHP) and disease-specific instruments (CAMPHOR) showed comparable HRQoL impairment in this patient population. CAMPHOR also showed a moderate impairment in overall QoL. WHO FC, 6MWD, and Borg dyspnoea index were highly correlated with HRQoL impairment in our cohort, as well as QoL measured through CAMPHOR. In our search for the “best” and “most” practical PROM to evaluate HRQoL and QoL in Portuguese PH patients other highly used general instruments and the newly developed disease-specific instruments will be tested.

## Figures and Tables

**Table 1:** Demographic and clinical characteristics of the study population.

Characteristics	PH patients (*n* = 49)
Age, years	50.4 ± 13.7
Gender, *n* (%)	
Female	37 (75.5)
Male	12 (24.5)
Marital status, *n* (%)	
Single	11 (22.4)
Married/divorced/widowed	38 (77.6)
Working status, *n* (%)	
Full-time	15 (34.7)
Homemaker	9 (18.4)
Retired	23 (46.9)
Disease duration, months	57.1 ± 58.8
PH aetiology, *n* (%)	
PAH and others	37 (75.5)
CTEPH	12 (24.5)
Comorbidities, *n* (%)	
Yes	28 (57.1)
No	21 (42.9)
WHO Functional class, *n* (%)	
I/II	34 (69.4)
III/IV	15 (30.6)
6MWD, meters	428.0 ± 105.8
Borg dyspnea	2.2 ± 2.6
HR_Bas, bpm	78.2 ± 11.6
HR_Max, bpm	107.9 ± 19.1
SBP, mmHg	113.4 ± 17.7
DBP, mmHg	68.2 ± 13.6
O2Sat_Bas, mmHg	94.0 ± 3.4
O2Sat_Min, mmHg	80.5 ± 15.1
NT-proBNP, pg/mL	684.6 ± 908.4
RAP, mmHg	7.1 ± 4.6
mPAP, mmHg	44.8 ± 18.2
PAOP, mmHg	9.8 ± 4.1
CI, L/min/m^2^	3.1 ± 0.9
PVR, Wood units	7.0 ± 4.3
Diuretics, *n* (%)	
Yes	29 (59.2)
No	20 (40.8)
Oral anticoagulants, *n* (%)	
Yes	24 (49.0)
No	25 (51.0)
Calcium channel blockers, *n* (%)	
Yes	5 (10.2)
No	44 (89.8)
Oxygen therapy, *n* (%)	
Yes	20 (40.9)
No	29 (59.1)
PH specific therapy, *n* (%)	
Monotherapy	23 (46.9)
Combination therapy	20 (40.8)
No therapy^*∗*^	6 (12.2)
PH specific therapy route, *n* (%)	
Oral	36 (73.5)
Parenteral	7 (14.3)

Data displayed as mean ± SD, except when otherwise indicated; 6MWD: 6-minute walk distance; NT-proBNP: N-terminal pro-brain natriuretic peptide; Borg: Borg dyspnea score; CI: cardiac index; CTEPH: chronic thromboembolic pulmonary hypertension; DBP: diastolic blood pressure; HR_Bas: baseline heart rate; HR_Max: maximum heart rate; mPAP: mean pulmonary arterial pressure; PAH: pulmonary arterial hypertension; PAOP: pulmonary artery occlusion pressure; PVR: pulmonary vascular resistance; RAP: right atrial pressure; O2Sat_min: minimum oxygen saturation; O2Sat_bas: baseline oxygen saturation; SBP: systolic blood pressure. ^*∗*^No therapy: CTEPH patients waiting for surgical treatment (*n* = 2); Porto-pulmonary hypertension waiting for liver transplantation (*n* = 1). Low risk congenital heart disease (*n* = 2), and idiopathic PAH before specific therapeutic introduction (*n* = 1).

**Table 2:** Correlation and multivariate linear regression for the relationship between patient characteristics and CAMPHOR scores.

Characteristics	Symptoms		Functioning		Quality of life	
	Correlation coefficient	Linear regression coefficient [95% CI]	Correlation coefficient	Linear regression coefficient [95% CI]	Correlation coefficient	Linear regression coefficient [95% CI]
Age, yrs	0.240		0.403^*∗∗*^		0.283^*∗*^	
Work status						
Full-time	−0.172		−0.360^*∗*^		−0.166	
Homemaker	0.095		0.002		0.064	
Retired	0.090		0.342^*∗*^		0.109	
Etiology						
PAH/others	0.328^*∗*^		0.350^*∗*^		0.333^*∗*^	
CTEPH						
Comorbidities						
Yes	0.297^*∗*^		0.228		0.206	
No						
Functional class						
I/II	0.526^*∗∗∗*^		0.627^*∗∗∗*^	-	0.505^*∗∗∗*^	
III/IV				4.74		
				[1.23; 8.26]		
6MWD, meters	−0.673^*∗∗∗*^	−0.03	−0.742^*∗∗∗*^	−0.02	−0.609^*∗∗∗*^	−0.03
		[−0.05; −0.01]		[−0.04; −0.01]		[−0.04; −0.01]
Borg dyspnea	0.779^*∗∗∗*^	1.29	0.652^*∗∗∗*^		0.732^*∗∗∗*^	1.19
		[0.64; 1.93]				[0.59; 1.78]
HR_bas, bpm	−0.209		−0.299^*∗*^	−0.13	−0.301^*∗*^	
				[−0.24; −0.02]		
HR_Max, bpm	−0.338^*∗*^		−0.411^*∗∗*^		−0.367^*∗*^	
O2Sat_min, mmHg	−0.372^*∗∗*^		−0.335^*∗*^		−0.295^*∗*^	
Delta O2Sat	0.392^*∗∗*^		0.358^*∗*^		0.333^*∗*^	
Oxygen use						
Yes	0.340^*∗*^		0.330^*∗*^		0.324^*∗*^	
No						
Constant	NA	19.11		26.60		16.71
		[11.3; 26.9]		[16.9; 36.3]		[9.5; 23.9]
*R* ^2^ adjusted	NA	0.595	NA	0.527	NA	0.592

For the purpose of brevity only variables with significant results are displayed in the table. The following variables were considered for statistical analysis but did not reach statistical significance: gender, marital status, disease duration, DeltaHR, SBP, DBP, O2Sat_baseline, NT-proBNP, RAP, mPAP, PAOP, CI, PVR, oral anticoagulants, diuretics, calcium channel blockers, PH-specific therapy, PH-specific therapy route; correlation coefficients calculated using Spearman's rank (quantitative versus quantitative variables) or point-biserial (quantitative versus categorical). *R*^2^ adjusted represents the proportion of variability explained by the proposed model; 6MWD: 6-minute walk distance; NT-proBNP: N-terminal pro-brain natriuretic peptide; Borg: Borg dyspnea score; CAMPHOR: Cambridge Pulmonary Hypertension Outcome Review; CI: cardiac index; CTEPH: Chronic thromboembolic pulmonary hypertension; DBP: Diastolic blood pressure; DeltaHR: maximum-baseline heart rate; Delta O2Sat: baseline-minimum oxygen saturation; HR_Bas: baseline heart rate; HR_Max: maximum heart rate; mPAP: mean pulmonary arterial pressure; NA: not applicable; NHP: Nottingham Health Profile; NS: not significant; PAH: pulmonary arterial hypertension; PAOP: pulmonary artery occlusion pressure; PVR: pulmonary vascular resistance; RAP: right atrial pressure; O2Sat_min: minimum oxygen saturation; O2Sat_bas: baseline oxygen saturation; SBP: systolic blood pressure; ^*∗*^*P* < 0.05; ^*∗∗*^*P* < 0.01; ^*∗∗∗*^*P* < 0.001.

**Table 3:** Correlation and multivariate linear regression for the relationship between patient characteristics and NHP scores.

Characteristics	NHP											
	Energy Level		Pain		Emotional reactions		Sleep disorders		Social isolation		Physical mobility	
	Correlation coefficient	Linear regression coefficient [95% CI]	Correlation coefficient	Linear regression coefficient [95% CI]	Correlation coefficient	Linear regression coefficient [95% CI]	Correlation coefficient	Linear regression coefficient [95% CI]	Correlation coefficient	Linear regression coefficient [95% CI]	Correlation coefficient	Linear regression coefficient [95% CI]
Age, yrs	0.241		0.233		0.049		0.115		0.128		0.300^*∗*^	
Gender												
Female	−0.155		−0.118		−0.216		−0.308^*∗*^	-	−0.241		0.197	
Male								−28.2				
								[−50.1; −6.3]				
Work status												
Full-time	−0.226		−0.328^*∗*^		−0.152		−0.239		−0.046		−0.201	
Homemaker	0.082		0.009		0.200		0.050		0.112		0.000	
Retired	0.146		0.303^*∗*^		−0.012		0.188		−0.044		0.188	
Disease duration, months	−0.134		−0.294^*∗*^		0.094		−0.179		−0.098		−0.239	
Etiology												
PAH/others	0.344^*∗*^		0.352^*∗*^	-	0.338^*∗*^		0.242		0.257		0.409^*∗∗*^	
CTEPH				27.2								
				[9.4; 45.1]								
Comorbidities												
Yes	0.295^*∗*^		0.207		0.116		0.098		0.172		0.310^*∗*^	
No												
Functional class												
I/II	0.686^*∗∗∗*^	-	0.555^*∗∗∗*^		0.309^*∗*^		0.166		0.382^*∗∗*^		0.649^*∗∗∗*^	-
III/IV		30.7										21.8
		[11.8; 49.6]										[7.60; 36.1]
6MWD, meters	−0.578^*∗∗∗*^	5.89	−0.507^*∗∗∗*^		−0.293^*∗*^		−0.189		0.291^*∗*^		−0.742^*∗∗∗*^	−0.12
		[2.66; 9.13]										[−0.18; −0.06]
Borg dyspnea	0.747^*∗∗∗*^		0.544^*∗∗∗*^		0.607^*∗∗∗*^	3.79	0.296^*∗*^		0.488^*∗∗∗*^	5.03	0.617^*∗∗∗*^	
						[2.19; 5.38]				[2.75; 7.31]		
HR_bas, bpm	−0.117		−0.187		−0.427^*∗∗∗*^	−0.42	−0.341^*∗*^		−0.406^*∗∗*^		−0.185	
						[−0.78; −0.07]						
HR_Max, bpm	−0.421^*∗∗∗*^		−0.410^*∗∗∗*^	−0.438	−0.174		−0.153		−0.214		−0.294^*∗*^	
				[−0.84; −0.04]								
DeltaHR	−0.346^*∗*^		−0.298		0.058		0.038		0.024		−0.202	
SBP, mmHg	−0.124		−0.039		−0.144		−0.187		−0.349^*∗*^		−0.041	
O2Sat_min, mmHg	−0.283		−0.230		−0.285		−0.025		−0.279		−0.353^*∗*^	
Delta O2Sat	0.323^*∗*^		0.271		0.284		0.032		0.280		0.361^*∗*^	
NT-proBNP, pg/mL	0.161		0.134		0.063		−0.094		−0.012		0.318^*∗*^	
Oxygen use												
Yes	0.242		0.182		0.204		0.057		0.222		0.328^*∗*^	
No												
PH specific therapy												
None	−0.029		0.053		0.057		0.104		−0.006		0.058	
Monotherapy	0.058		0.104		0.012		0.268		−0.018		−0.011	
Combination therapy	−0.040		−0.143		−0.051		−0.343^*∗*^		0.022		−0.028	
Constant	NA		NA	58.3	NA	42.3	NA	35.4	NA		n.a.	71.1
				[14.1; 102.4]		[13.5; 71.0]		[24.7; 46.1]				[42.5; 99.8]
Adjusted *R*^2^	NA	0.504	NA	0.218	NA	0.441	NA	0.113	NA	0.290	n.a.	0.602

For the purpose of brevity only variables with significant results are displayed in the table. The following variables were considered for statistical analysis but did not reach statistical significance: marital status, DBP, DeltaBP, Sat_bas, RAP, mPAP, PAOP, CI, PVR, oral anticoagulants, diuretics, calcium channel blockers, PH-specific therapy route; correlation coefficients calculated using Spearman's rank (quantitative versus quantitative variables) or point-biserial (quantitative versus categorical). *R*^2^ adjusted represents the proportion of variability explained by the proposed model; 6MWD: 6-minute walk distance test; NT-BNP: N-terminal pro-brain natriuretic peptide; Borg: Borg dyspnea score; CAMPHOR: Cambridge Pulmonary Hypertension Outcome Review; CI: cardiac index; CTEPH: chronic thromboembolic pulmonary hypertension; DBP: diastolic blood pressure; DeltaHR: maximum-basal heart rate; DeltaSat: basal-minimum oxygen saturation; HR_Bas: basal heart rate; HR_Max: maximum heart rate; mPAP: mean pulmonary arterial pressure; NA: not applicable; NHP: Nottingham Health Profile; NS: not significant; PAH: pulmonary arterial hypertension; PAOP: pulmonary artery occlusion pressure; PVR: pulmonary vascular resistance; RAP: right atrial pressure; Sat_min: minimum oxygen saturation; Sat_bas: basal oxygen saturation; SBP: Systolic blood pressure; ^*∗*^*P* < 0.05; ^*∗∗*^*P* < 0.01; ^*∗∗∗*^*P* < 0.001.

## Supplementary Materials

Supplementary Materials Figure S1: mean CAMPHOR and NHP scores according to gender. Figure S2: mean CAMPHOR and NHP scores according to use of oxygen therapy. Figure S3: mean CAMPHOR and NHP scores according to PH aetiology. Figure S4: mean CAMPHOR and NHP scores according to type of PH-specific therapy. Figure S5: scatterplots for high correlations between CAMPHOR/NHP scores and 6MWD. Figure S6: scatterplots for high correlations between CAMPHOR/NHP scores and Borg dyspnoea.
