# Supplementary material for: Health-Related Quality of Life in Pulmonary Hypertension and Its Clinical Correlates: A Cross-Sectional Study
Source: Biomed Res Int. 2018 Mar 19;2018:3924517. doi: 10.1155/2018/3924517 (PMC5884279; doi:10.1155/2018/3924517)

**Supplementary material**

**Table of contents:**

**Figure S1.** Mean CAMPHOR and NHP scores according to Gender.

**Figure S2.** Mean CAMPHOR and NHP scores according to use of Oxygen therapy.

**Figure S3.** Mean CAMPHOR and NHP scores according to PH aetiology.

**Figure S4.** Mean CAMPHOR and NHP scores according to type of PH-specific therapy.

**Figure S5.** Scatterplots for high correlations between CAMPHOR/NHP scores and 6MWD.

**Figure S6.** Scatterplots for high correlations between CAMPHOR/NHP scores and Borg Dyspnoea.

**Figure S1.** Mean CAMPHOR and NHP scores according to Gender.


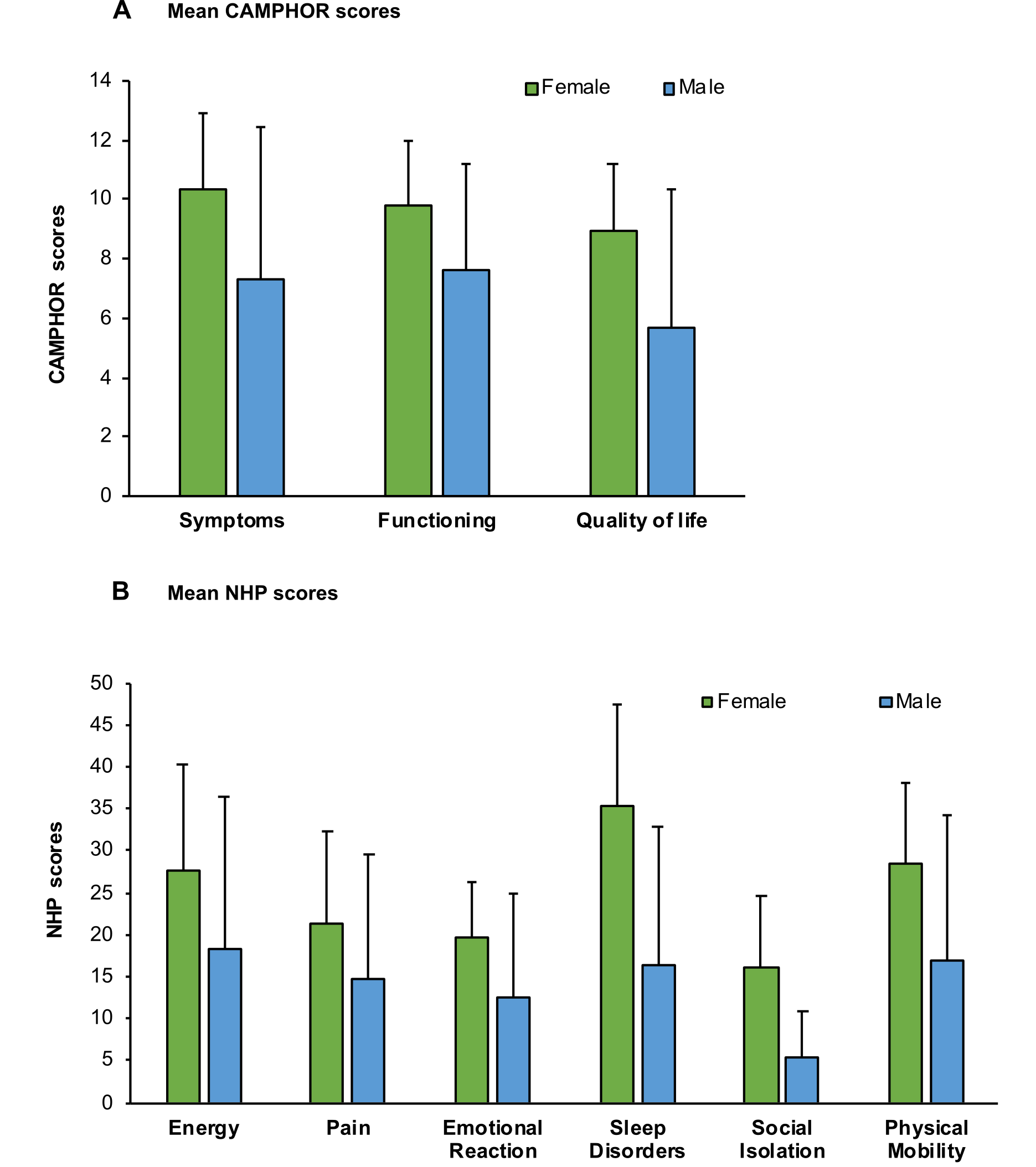


CAMPHOR: Cambridge Pulmonary Hypertension Outcome Review; NHP: Nottingham Health Profile
Error bars represent 95% confidence intervals.

**Figure S2.** Mean CAMPHOR and NHP scores according to use of Oxygen therapy.


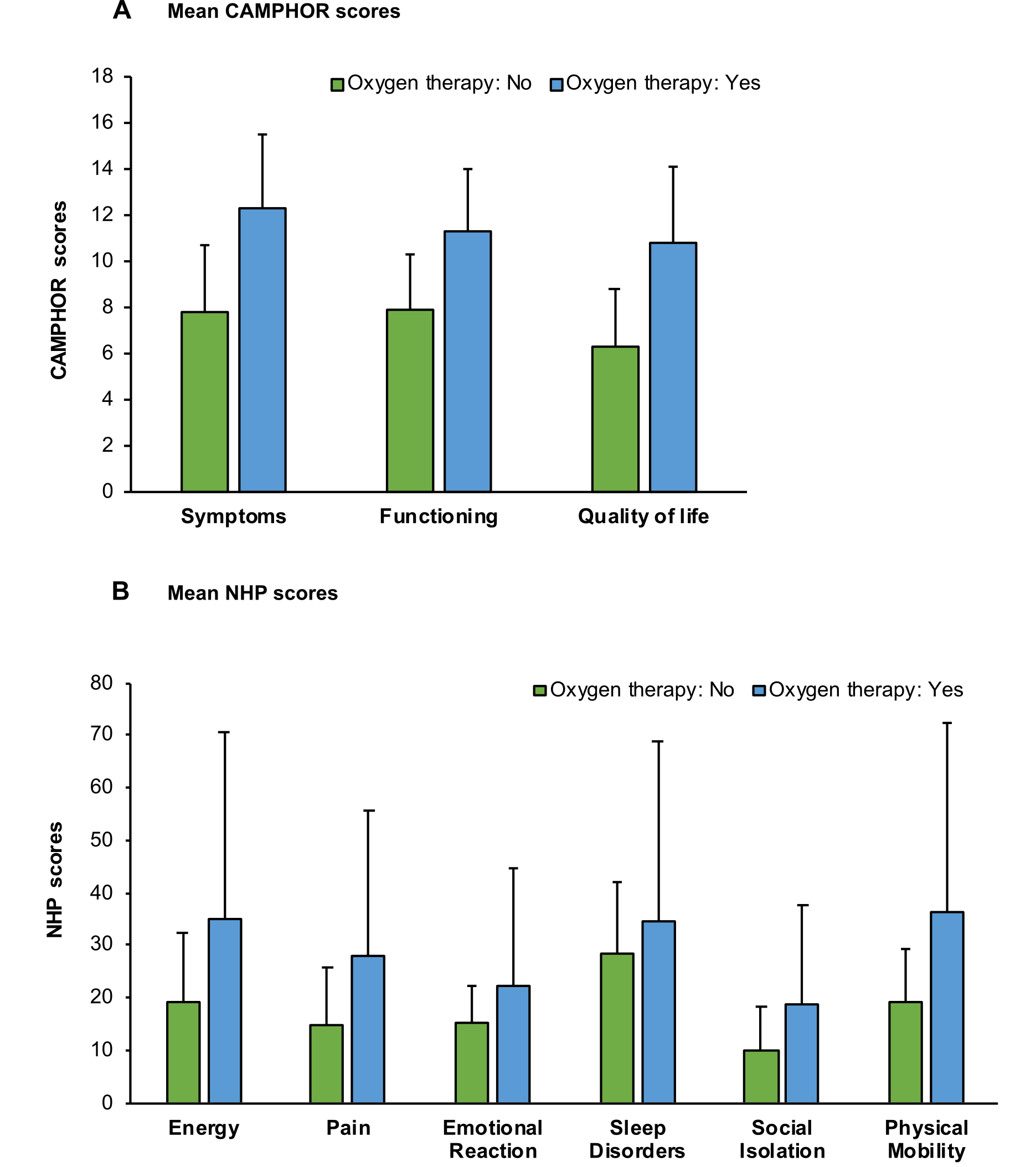


CAMPHOR: Cambridge Pulmonary Hypertension Outcome Review; NHP: Nottingham Health Profile
Error bars represent 95% confidence intervals.

**Figure S3.** Mean CAMPHOR and NHP scores according to PH aetiology.


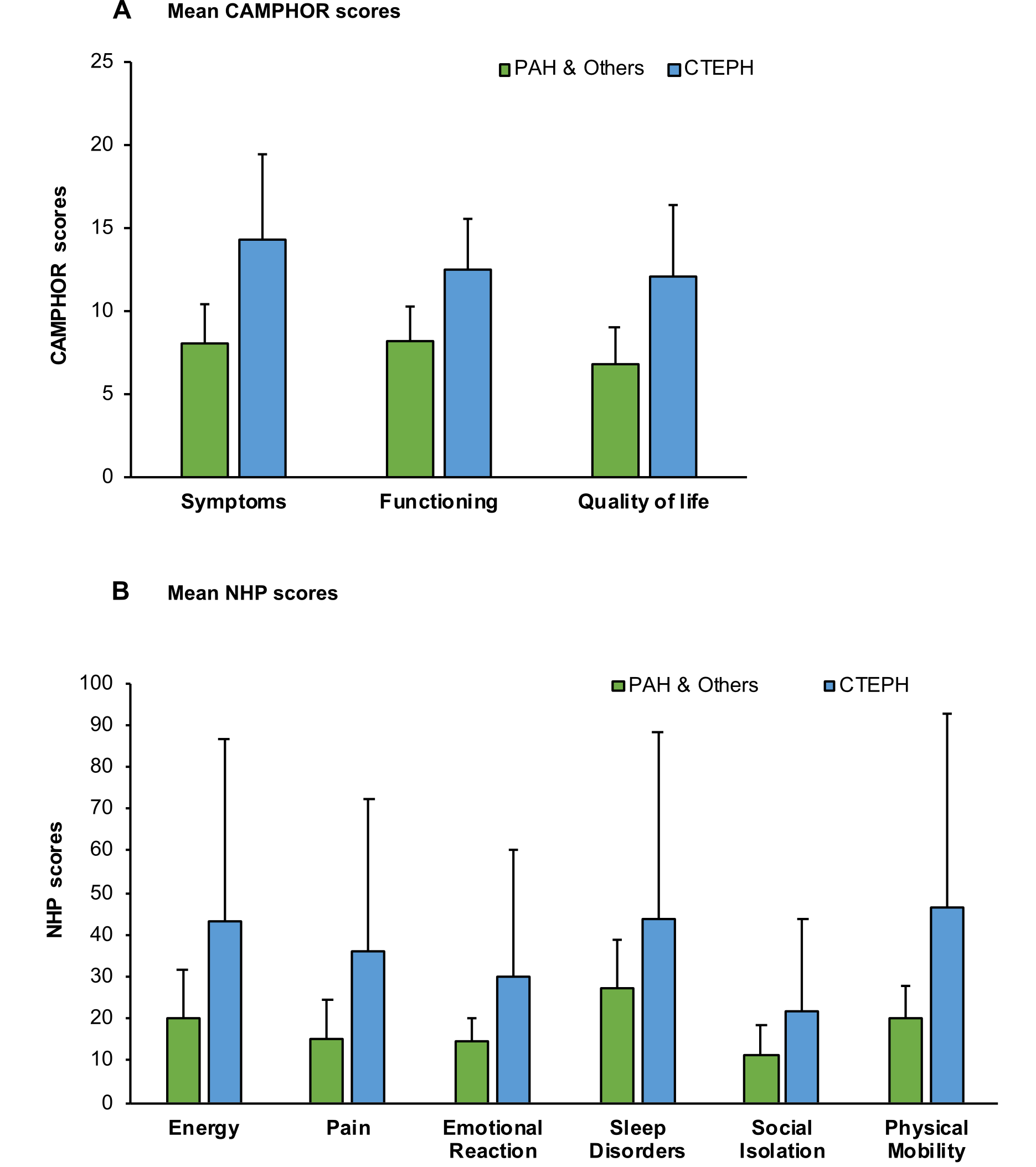


CAMPHOR: Cambridge Pulmonary Hypertension Outcome Review; NHP: Nottingham Health Profile; PH: Pulmonary Hypertension Error bars represent 95% confidence intervals.

**Figure S4.** Mean CAMPHOR and NHP scores according to type of PH-specific therapy.


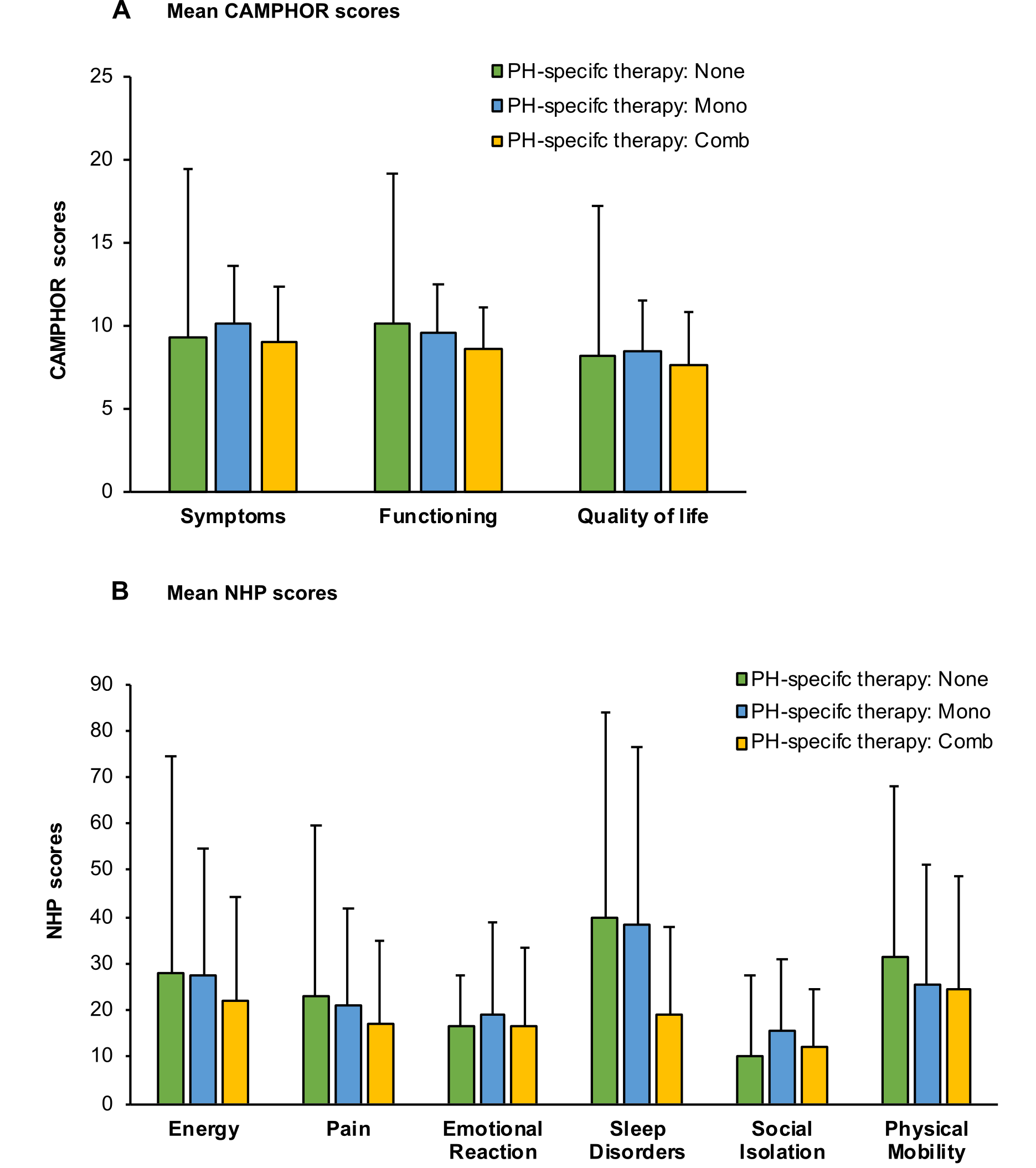


CAMPHOR: Cambridge Pulmonary Hypertension Outcome Review; NHP: Nottingham Health Profile; PH: Pulmonary Hypertension
Error bars represent 95% confidence intervals.

**Figure S5.** Scatterplots for high correlations between CAMPHOR/NHP scores and 6MWD.


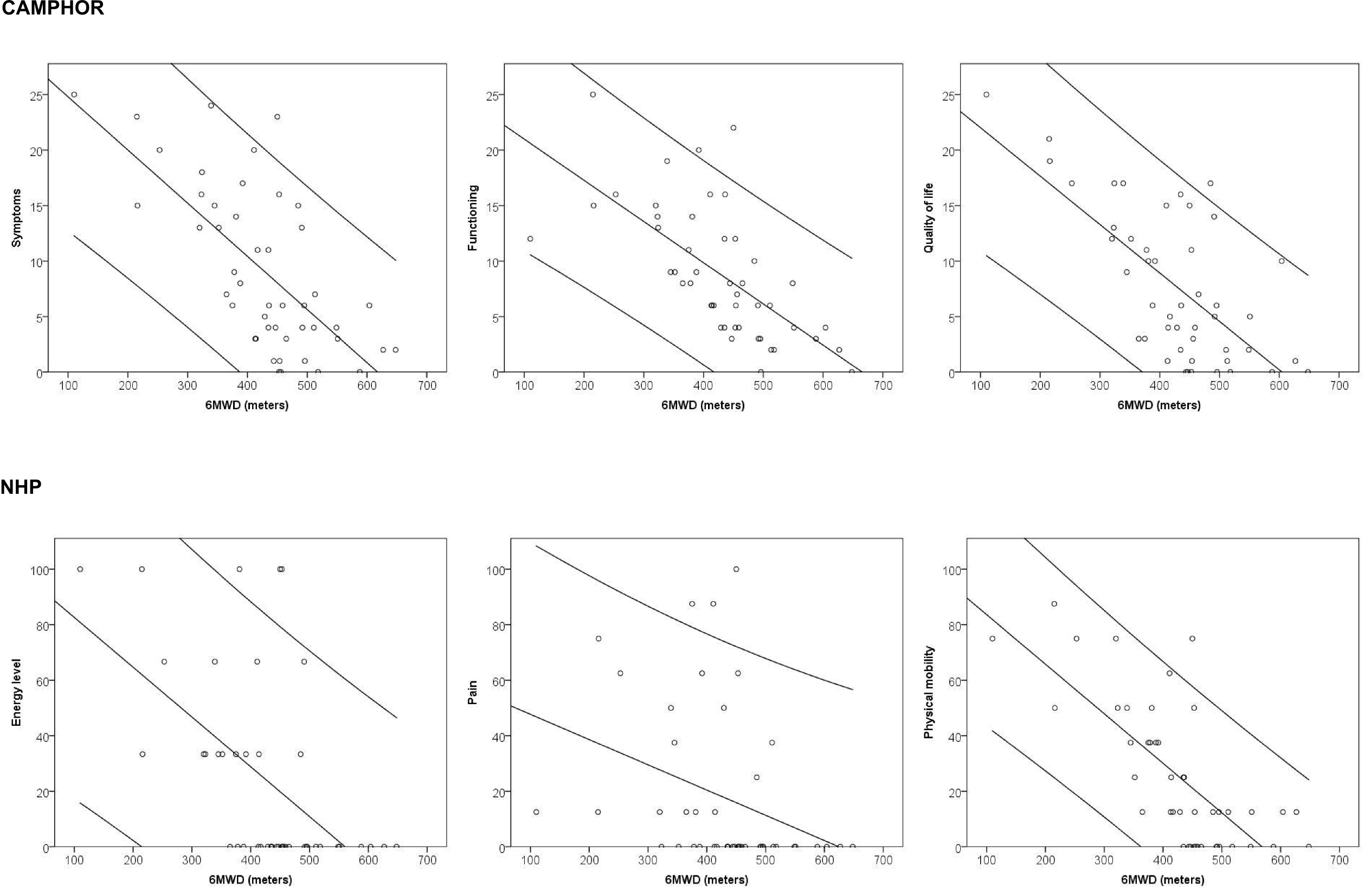


6MWD: 6-minute walking distance; CAMPHOR: Cambridge Pulmonary Hypertension Outcome Review; NHP: Nottingham Health Profile; PH: Pulmonary Hypertension

**Figure S6.** Scatterplots for high correlations between CAMPHOR/NHP scores and Borg Dyspnoea.


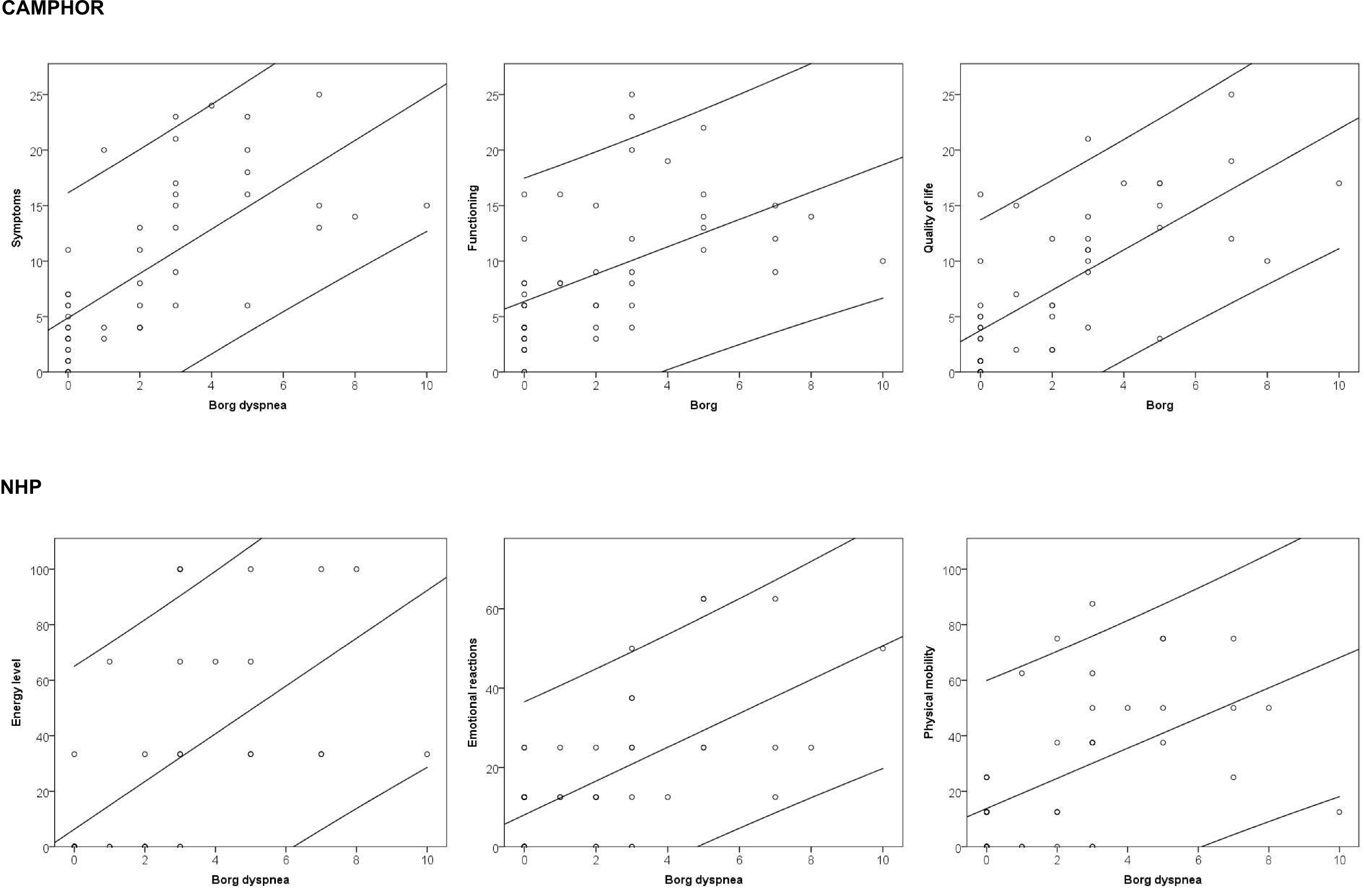

Supplement: Supplementary Materials — Figure S1: mean CAMPHOR and NHP scores according to gender. Figure S2: mean CAMPHOR and NHP scores according to use of oxygen therapy. Figure S3: mean CAMPHOR and NHP scores according to PH aetiology. Figure S4: mean CAMPHOR and NHP scores according to type of PH-specific therapy. Figure S5: scatterplots for high correlations between CAMPHOR/NHP scores and 6MWD. Figure S6: scatterplots for high correlations between CAMPHOR/NHP scores and Borg dyspnoea. [file 3924517.f1.docx]
